# Anti-Inflammatory and Antiapoptotic Responses to Infection: A Common Denominator of Human and Bovine Macrophages Infected with *Mycobacterium avium* Subsp. *paratuberculosis*

**DOI:** 10.1155/2013/908348

**Published:** 2013-01-20

**Authors:** Naiara Abendaño, Ramon A. Juste, Marta Alonso-Hearn

**Affiliations:** Department of Animal Health, Basque Institute for Agricultural Research and Development, NEIKER-Tecnalia, Technological Park of Bizkaia, 48160 Derio, Bizkaia, Spain

## Abstract

*Mycobacterium avium* subsp. *paratuberculosis* (*Map*) is the causative agent of a chronic intestinal inflammation in ruminants named Johne's disease or paratuberculosis and a possible etiopathological agent of human Crohn's disease (CD). Analysis of macrophage transcriptomes in response to *Map* infection is expected to provide key missing information in the understanding of the role of this pathogen in establishing an inappropriate and persistent infection in a susceptible host and of the molecular mechanisms that might underlie the early phases of CD. In this paper we summarize transcriptomic studies of human and bovine peripheral blood mononuclear cells (PBMC), monocyte-derived macrophages (MDMs), and macrophages-like cell lines *in vitro* infected with *Map*. Most studies included in this paper consistently reported common gene expression signatures of bovine and human macrophages in response to *Map* such as enhanced expression of the anti-inflammatory cytokines IL-10 and IL-6, which promote bacterial survival. Overexpression of IL-10 could be responsible for the *Map*-associated reduction in the expression of the proapoptotic TNF-**α** gene observed in bovine and human macrophages.

## 1. Association of *Mycobacterium avium *Subsp. *paratuberculosis (Map)* with Chronic Inflammatory Bowell Diseases of Cattle and Humans

*Mycobacterium avium* subsp. *paratuberculosis *(*Map*) is the causal agent of Johne's disease or paratuberculosis, a chronic inflammatory bowel disease of domesticated ruminants and wildlife species worldwide. Johne's disease causes major economic losses to the global dairy industry due to reduced milk production, lower weight gains, infertility, premature culling, and increased cow replacement costs [1]. *Map *has a worldwide distribution and is of considerable concern in cattle, sheep, goats, and farmed red deer. Although it is still controversial, *Map* has been implicated as a causal or exacerbating agent in human Crohns's disease (CD), a chronic inflammatory bowel disease characterized by transmural inflammation and granuloma formation [2–4]. Evidences that *Map *may be associated to CD in humans include similarity between the clinical signs of CD in humans and those found in animals with paratuberculosis; detection of *Map* in feces, intestinal tissues, breast milk, macrophages, and peripheral blood of patients with CD; association between *Map* DNA in blood and cellular and humoral immune responses in CD; and anti-*Map *antibiotic therapy resulting in reduction of bacteremia and remission or substantial improvement in disease condition in many patients [5–10]. In addition, meta-analysis and epidemiological studies have confirmed an association of *Map* with CD [11, 12]. 

*Map* may enter the food chain from a variety of sources. The organism, shed from infected animals, may contaminate pastures and potable water, where it is resistant to standard purification with chlorine [13]. Because *Map* can survive pasteurization conditions, dairy products such as milk and cheese have been proposed as possible sources of exposure of humans to *Map* [14]. Recently, we have demonstrated that *Map *can be detected and cultured from diaphragm muscle of *Map*-infected cattle destined for human consumption and suggested a possible risk of exposure of humans to *Map* via contaminated meat [15]. After oral ingestion, *Map* invades the intestinal wall preferably through epithelial cells or M cells present in the follicle-associated epithelium covering the continuous Peyer's patches in the distal ileum [16–18]. Although the mechanism of entry in the mucosa is important in establishing *Map *infection, most of the bacterial components involved in the interaction with the intestinal epithelium are still unknown. It has been recently demonstrated that *Map*3464 gene encodes an NADH-flavin oxidoreductase involved in invasion of bovine epithelial cells through the activation of host cell Cdc42 [19]. After translocation of *Map* across the intestinal epithelium, *Map* is subsequently phagocytised by macrophages in the intestinal lamina propria and submucosa. Upon phagocytosis of *Map* by naive macrophages, there is both intracellular replication of *Map *and bacterial killing by the host, which reflects an initial T-helper 1 (Th-1) or proinflammatory immune response [20–24]. Bacterial killing is due to a rapid phagosome acidification response by the host that enables phagosome-lysosome fusion and presentation of antigens to T cells via MHC to occur in some infected cells. However, since many phagosomes containing *Map *fail to acquire significant amounts of lysosomal-associated membrane protein (LAMP-1) and to fuse with lysosomes, this can allow *Map* to survive and proliferate inside macrophages. An active role for *Map *in preventing phagosome-lysosome fusion is supported by the observation that live bacteria are able to persist within phagosomes, while phagosome maturation is not interrupted following the uptake of killed *Map* [25, 26]. In addition, recent studies have suggested that *Map *alters the ability of infected macrophages to react to extracellular signals from T cells, particularly through the CD154-CD40 system [27]. This leads to an enhanced IL-10 and TGF-*β* expression in *Map*-infected macrophages, which favor bacterial survival by suppression of Th-1 responses and IFN-*γ* in T cells [28–31]. In cattle, clinical signs of infection and bacterial shedding are usually not evident until 2–5 years post-infection (p.i.) [32]. During the subclinical phase of the infection, *Map* persists and slowly proliferates within macrophages of the gut without the innate system being able to clear the infection. 

## 2. Anti-Inflammatory, Antiapoptotic, and Anti-Invasive Responses Induced in Bovine Macrophages Infected with *Mycobacterium avium* Subsp. *paratuberculosis*

Several *in vitro* studies have investigated gene expression profiles induced by *Map* on bovine macrophages obtained from uninfected cattle and on a bovine macrophage cell line (Table 1). Compared to uninfected cultures, *in vitro* challenges of monocytes-derived macrophages (MDMs) from healthy cows with live *Map* resulted in enhanced production of the anti-inflammatory cytokine interleukin-10 (IL-10) at 6, 24, and 72 h p.i. that antagonizes the proinflammatory immune response by downregulating the production of interleukin-12 (IL-12), tumor necrosis factor-*α* (TNF-*α*), and interferon-*γ* (IFN-*γ*) as estimated by qRT-PCR [33]. Similarly, other authors also observed a downregulatory trend in TNF-*α* mRNA expression from 16 h to 96 h p.i. and upregulation of IL-10 mRNA levels that peak from 48 h to 96 h p.i. [34]. Using microarray technology, three cytokines including transforming growth factor-*β* (TGF-*β*), interleukin-6 (IL-6), and macrophage inflammatory protein-1*β* (MIP-1*β*) had greater expression in *Map*-infected MDM at 16 h p.i. when compared with inactivated control macrophages [35]. The matrix metalloproteinase (MMP-12) and the thrombospondin-1, both involved in cell migration and tissue destruction, were also significantly upregulated. In contrast, the TNF-*β* receptor and the major histocompatibility complex (MHC) class II DQ-*β* had lower expression in *Map*-infected macrophages. Decreased expression of the cell surface MHC class I and class II molecules was previously documented in macrophages phagocytising *Map *organisms indicating a reduced capacity to present antigens to T lymphocytes [44]. Consistently with these results, Murphy et al. also detected high levels of the anti-inflammatory cytokines TGF-*β* and IL-6 in MDM infected with *Map* at 24 h p.i. [36]. In another study, significantly downregulation in expression of the proinflammatory cytokine IL-1 and of the metalloproteinases MMP-1, MMP-23, and MMP-9 involved in tissue destruction was observed in *Map*-stimulated PBMC when compared with control cells [37]. Using cDNA microarrays focused on expressed sequences from a bovine total leukocyte library (BOLT5) and 10 distinct *Map* strains to measure total transcriptomic alterations in *Map*-infected MDM, a total of 78 annotated bovine genes were found to be differentially expressed at 6 h p.i., relative to uninfected cells [38]. Within the group of differentially expressed genes significant downregulation of two proapoptotic genes, BCL2 antagonist of cell death (BAD) and TNF receptor (TNFR), was observed in *Map*-infected MDM cells relative to uninfected cells. Upregulation of the apoptotic inhibitor BCL2A1 and of the proinflammatory cytokines IL-1*α*, IL-1*β*, and IL-8 relative to uninfected control cells was also observed. By using a pan-genomic analysis of bovine MDM gene expression in response to *in vitro* infection with *Map*, Machugh et al. revealed that many of the highly upregulated genes at 2 h p.i. had proinflammatory related functions, particularly IL-1*α*, IL-1*β*, TNF, IL-6, chemokine ligand 2 gene (C-X-C motif; CXCL2), and the chemokine ligand 20 gene (C-C motif; CCL20) [39]. At 6 h p.i. immune-related genes were among the differentially expressed genes showing the highest relative increase in expression; however, the fold-change induction of these genes was not as high as those detected as 2 h p.i. Upregulated genes at 6 h p.i. included IL-1*β*, TNF, CXCL2, CCL4, CCL5, CCL20, CD40, and the complement factor B gene (CFB). Of the differentially expressed gene identified 24 h p.i. that had a known immune function were the serum amyloid A3 genes (SAA3), C-type lectin domain family 4 member E (CLEC4E), C-type lectin domain family 2 member D (CLEC2D), CD40, and CFB. Overall, several pro- and antiapoptotic genes were upregulated at 2 h and 6 h p.i. suggesting that this process is highly regulated. Pro- and antiapoptotic genes upregulated included TNF (proapoptotic), caspase 1, 4, 6, and 8 genes (CASP-1, CASP-4, CASP-6, and CASP-8; all proapoptotic), the baculoviral IAP repeat containing 3 gene (BIRC-3; antiapoptotic), and the FADD-like apoptosis regulator gene (CFLAR; pro- and antiapoptotic).

We recently examined whether *Map *isolates with differential abilities to grow within a bovine macrophage cell line (BoMac) induced a characteristic early immune-inflammatory response [40]. Our results showed significant differences in the expression of several cytokines (IL-6, TGF-*β*1, TNF-*α*2, IFN-*γ*, and IL-1*α*), proteins related to apoptosis (BCL2-1), or tissue destruction (MMP3-1) after the infection of BoMac cells with a bovine or an ovine isolate of *Map*. The bovine isolate that grew within BoMac cells was a good inducer of the apoptotic inhibitor BCL2-1 at 4 or 14 h p.i. which might cause lower levels of apoptosis than in BoMac cells infected with the ovine isolate. In addition, infection of BoMac cells with the bovine isolate resulted in a significant upregulation of the anti-inflammatory cytokines IL-6 and TGF-*β*1 at 24 h p.i., when compared with cells infected with the ovine isolate. Although we did not observe significant differences in IL-10 or TNF-*α*2 gene expression in BoMac cells infected with the bovine or the ovine isolates at any of the time points, the bovine isolate did induce more IL-10 at 14 and 24 h p.i and less TNF-*α*2 at 4 and 14 h p.i. than did the ovine isolate. These results suggest that lower TNF-*α*2 production and an induction of IL-10 are associated with the growth within bovine macrophages of virulent isolates of *Map*. We also observed that cells stimulated with the bovine isolate exhibited lower levels of the metalloproteinase MMP3-1 involved in tissue destruction at 4 h and 24 h p.i. relative to cells stimulated with the ovine isolate. Differences in induction of the metalloproteinase inhibitor TIMP-1 were not statistically significant at any of the time points studied but the bovine isolate did induce more TIMP-1 at 4 h p.i. than did the ovine isolate. The ovine isolate was significantly attenuated in growth in BoMac cells and this decrease in survival within the infected cells correlated with a reduced anti-inflammatory response in the infected cells and with a significantly upregulated proinflammatory immune response generally associated with elimination of *Map *and protection. In particular, the expression of the proinflammatory cytokine IL-1*α* was highly upregulated in cells infected with the ovine isolate at 14 and 24 h p.i. and downregulated in BoMac cells infected with the bovine isolate at 24 h p.i. Because a strong correlation between the intracellular multiplication of the tested isolates and patterns of production of IL-6, TGF-*β*, MMPL-3, BCL2-1, and IL-1*α* was observed, the levels of expression of these specific proteins might be used to discriminate between isolates with differential virulence in the BoMac cellular model.

All together, the results of the transcriptomic studies in bovine macrophages included in this paper suggested that *Map* might stimulated an initial proinflammatory immune response mediated by IL-1*α* that is followed by an enhanced anti-inflammatory response mediated by IL-6, IL-10, and TGF-*β*. In addition, downregulation of the proapoptotic gene TNF-*α* was consistently observed in different studies. Stimulation of anti-inflammatory and antiapoptotic responses might allow *Map* to successfully persist in bovine macrophages during the persistent, subclinical phase of the infection.

## 3. Immune-Inflammatory Responses Induced in Human Macrophages Infected with **Map**

Despite the possible role of *Map* in CD, there is not much known about the interaction of *Map* with the human innate immune system (Table 2). A transformed human monocytic cell line (THP1) stimulated with *Map* has shown to differentially respond to infections with well-characterized clinical isolates of *Map* when compared with unstimulated cells. When human THP1 cells were stimulated with bovine or human isolates of *Map* several genes associated with apoptosis and cytokine signalling (LRDD, PDCD-8, IL-12, IL-18, IL-23, and TNF*α*) were significantly downregulated [41]. Data from this study suggested that the human macrophage responses to *Map* isolates from cattle or human sources, regardless of genotype, follow a common theme of antiapoptotic and anti-inflammatory responses within the host cells, an attribute likely associated with successful infection and persistence.

 The response of macrophages from CD patients to live *Map *has been recently addressed. Following *in vitro* exposure to *Map*, PBMC from CD patients secreted significant more amounts of TNF-*α*, IL-6, and IL-10 when compared to the levels released by PBMC from healthy volunteers [42]. In contrast, the IFN-*γ* response to *Map* was significantly elevated in PBMC isolated from healthy volunteers compared to PBMC derived from CD patients. In another study, human MDMs obtained from CD patients and controls were infected with *Map*, *Mycobacterium avium* subsp.* avium* (*Mav*), and other live intestinal bacteria such as *Escherichia coli *or *Enterococcus faecalis*, and cytokine levels were evaluated at different time points [43]. The results of this study indicated that macrophages from CD patients showed impaired TNF-*α* secretion in response to bacterial challenge but augmented IL-23 secretion as compared to macrophages from healthy individuals. It is plausible that the MAP-dependent IL-23 secretion enhancement occurred as consequence of phagocytosis, because the high IL-23 concentrations obtained at 3 h p.i. were not observed at 3 d and 7 d p.i. Differences in cytokine expression after bacterial challenge where not *Map* specific, as other bacteria (*E. coli* and *Mav*) showed similar effects.

Although most of the studies presented in this paper used PBMC or MDM cells *in vitro *stimulated with *Map, *we should indicate that recently Olsen et al. [45] isolated intestinal T cells from intestinal biopsies of CD patients to investigate cellular immune responses to *Map*. Interestingly, they observed that CD patients had a high frequency of *Map *reactive T cells and also a higher frequency of response to *Map *compared to other bacterial antigens. After stimulation with *Map*, intestinal T cells secreted the proinflammatory cytokines IFN-*γ* and IL-17, and, therefore, a role for *Map* in the excessive inflammation seen in CD cannot be excluded.

## 4. Conclusions 

Despite development of cell-mediated immune responses shortly after infection, *Map* has the capacity to survive and grow in macrophages from human and cattle hosts. Gene expression studies included in this paper allow us to conclude that the inhibition of apoptosis and enhanced expression of inhibitors of macrophage activation could contribute to the early survival and immune escape of *Map*. Common gene expression signatures of bovine and human macrophages in response to *Map* such as enhanced expression of the anti-inflammatory cytokines IL-10 and IL-6, which promote bacterial survival, have been consistently observed. Overexpression of IL-10 could be responsible for the *Map*-associated reduction in the expression of the proapoptotic TNF-*α* gene observed in many studies. Differential effects on macrophage gene regulation between studies might be caused by the different *in vitro* models, multiplicities of infection, time p.i., and/or microarray platforms used. Although this paper does not suggest a casual effect for *Map*, it does compile the findings that *Map *is able to alter the normal host immune response against a pathogen in susceptible humans and cattle and contribute to the pathogenesis of Crohn's and Johne's diseases.

## Figures and Tables

**Table 1:** Immune related, apoptosis-related and tissue destruction genes differentially expressed relative to uninfected cells in monocytes-derived macrophages (MDM), peripheral blood mononuclear cells (PBMC), and in a bovine macrophage cell line after stimulation with live bovine isolates of *Map*. Genes upregulated or downregulated in at least three of the studies are shown in bold.

Cell model	*Map* strain	*Map*/cells ratio	Assay	Time p.i. (h)	Upregulated genes	Downregulated genes	Reference
MDM^1^	ATCC 19698	10 : 1	qRT-PCR	6 h	**IL-10,** GM-CSF	IFN-*γ*, **TNF-*α***	[33]
				24 h	**IL-10**, GM-CSF		
				72 h	**IL-10**, GM-CSF, and IL-8	IL-12	

MDM	Field strain B1018	5 : 1	qRT-PCR	16 h		IL-10, **TNF-*α***	[34]
				24 h		IL-10, **TNF-*α***	
				48 h	**IL-10**	**TNF-*α***	
				96 h	**IL-10**	**TNF-*α***	

MDM	ATCC 19698	10 : 1	Microarray	16 h	**IL-6, TGF-*β***,	**TNFR,**	[35]
					Thrombospondin-1, MIP-1*β*, and MMP-12	MHC class II DQ-*β*	

MDM	ATCC 19698	10 : 1	Microarray	24 h	**IL-6, TGF-*β***		[36]

PBMC^2^	ATCC 19698	10 : 1	Microarray	16 h		IL-1, MMP1, MMP23, and MMP9	[37]

MDM	ATCC 19698	5 : 1	Microarray	6 h	IL-1*β*, IL-8, **IL-1*α***, and BCL2A1	**TNFR,** BAD	[38]

MDM	Field strain	2.1	Microarray	2 h	**IL-10, IL-6, IL-1*α***, IL-1*β*, TNF, CXCL2, CCL20, CFB, CASP-1, CASP-4, CASP-8, CASP-6, BIRC-3, CFLAR, and CD40		[39]
				6 h	IL-1*β*, TNF, CXCL2, CCL4, CCL5, CCL20, CD40, CFB, CASP-1, CASP4, CASP8, CASP6, BIRC3, and CFLAR		
				24 h	SAA3, CLEC4E, CLEC2D, CD40, and CFB		

BoMac^3^	K10	10 : 1	qRT-PCR	4 h	IFN-*γ*, **IL-1*α***, BCL2-1, and **TGF-*β*** **1**	IL-6	[40]
				14 h	**IL-6, **BCL2-1		
				24 h	**IL-6**,** TGF-*β*** **1**, and TNF*α*-2	MMP3-1, IL-1*α*, and BCL2-1	

^1^MDM: monocytes-derived macrophages; ^2^PBMC: peripheral blood mononuclear cells; ^3^BoMac: bovine macrophage cell line.

**Table 2:** Cytokine, apoptosis-related, and tissue destruction proteins differentially expressed in human transformed macrophage cell lines, in peripheral blood mononuclear cells (PBMC), and monocytes-derived macrophages (MDM) obtained from Crohn's disease (CD) patients after stimulation with bovine or human isolates of *Map*.

Cell Model	*Map* strain	*Map*/cells ratio	Assay	Time p.i. (h)	Upregulated cytokines	Downregulated cytokines	Reference
THP-1^1,2^	Bovine 1018	5 : 1	Microarray	2 h		IL-18, IL-12B, IL-23A, **TNF-*α*,** LRDD, and PDC28	[41]

PBMC from CD patients^3^	Human ATCC 43019	1 : 1	Flow cytometry	72 h	**IL-10, IL-6,** and TNF-*α*	IFN-*γ*	[42]

MDM from CD patients^3^	Human ATCC 43015	10 : 1	Flow cytometry	3 h 72 h	IL-23	**TNF-*α***	[43]

^1^THP-1, human monocytic cell line.

^2^Comparisons were made between uninfected and infected cells.

^3^Comparisons were made between *Map*-infected macrophages from CD patients and controls.
